# Detection of complicated ectopic pregnancies in the hospital discharge database: A validation study

**DOI:** 10.1371/journal.pone.0217674

**Published:** 2019-06-05

**Authors:** Marion Fermaut, Arnaud Fauconnier, Aurélie Brossard, Jimmy Razafimamonjy, Xavier Fritel, Annie Serfaty

**Affiliations:** 1 Department of Gynecology and Obstetrics, Intercommunal Hospital Centre of Poissy-Saint-Germain-en-Laye, Poissy, France; 2 EA 7285, Research Unit "Risk and Safety in Clinical Medicine for Women and Perinatal Health", Versailles-Saint-Quentin University (UVSQ), Montigny-le-Bretonneux, France; 3 Department of Gynecology and Obstetrics, University Hospital Center of Poitiers, Poitiers, France; 4 Medical Information Department, Intercommunal Hospital Centre of Poissy-Saint-Germain-en-Laye, Poissy, France; 5 INSERM CIC 1402, University Hospital Center of Poitiers, Poitiers, France; 6 Medical Information Department, Armand-Trousseau, La Roche-Guyon, Eastern Parisian University Hospital, Assistance Publique–Hôpitaux de Paris (AP-HP), Paris, France; 7 Regional Agency of Health for Paris Region, Direction of health promotion and inequality reduction, Paris, France; University of Oxford, National Perinatal Epidemiology Unit, UNITED KINGDOM

## Abstract

**Objective:**

Complicated ectopic pregnancies with severe bleeding (CEPSB) are life-threatening situations and should be considered maternal near-miss cases. Previous studies have found an association between severe maternal morbidity secondary to CEPSB and substandard care. Almost all women with CEPSB are hospitalized, generating administrative and medical records. The objective of this study was to propose a method to measure the validity of the hospital discharge database (HDD) to detect CEPSB among hospital stays in two gynecological units.

**Methods:**

We included all hospital stays of women who were 18–45 years old and hospitalized for acute pelvic pain or/and metrorrhagia in the two hospitals. The HDD was compared to medical data (gold standard). Two algorithms constructed from the International Classification of Disease (ICD-10) and Common Classification of Medical Procedures (CCAM), were applied to the HDD: a “predefined algorithm” according to coding guidelines and a “pragmatic algorithm” based on coding practices. Sensitivity, specificity and positive likelihood-ratios were calculated. False negatives and positives were analyzed to describe coding practices.

**Results:**

Among 370 hospital stays included, 52 were classified as CEPSB cases. The “predefined algorithm” gave a sensitivity of 23.1% (95% CI: 11.6–34.5) and a specificity of 99.1% (95% CI: 98.0–100.0) to identify CEPSB. The “pragmatic algorithm” gave a sensitivity of 63.5% (95% CI: 50.4–76.5) and a specificity of 94.7% (95% CI: 92.2–97.5) to identify CEPSB. Coding errors (77.6%) were due to misuse of diagnosis codes and because complications were not coded.

**Conclusion:**

HDD is not reliable enough to detect CEPSB due to incorrect coding practices. However, it could be an ideal tool to monitor quality of care if a culture in data quality assessment is developed to improve quality of medical information.

## Introduction

Ectopic pregnancies (EPs) account for approximately 2 to 3% of pregnancies each year [1–5]. Tubal rupture occurs in 22 to 34% of cases and often results in hemoperitoneum [2]. The Eighth Report of the Confidential Enquiries into Maternal Deaths in the United Kingdom, covering the period from 2006 to 2008, estimated a fatality rate of 16.9 (95% CI 7.6–37.6) per 100 000 EPs [6]. Furthermore, a study from the United States estimated EP to be the most common cause of mortality during the first trimester of pregnancy [7]. Thus, complicated EPs with severe bleeding (CEPSB) are life-threatening situations and should be considered maternal near-miss cases. Reducing the incidence of complications of EP is a national public health goal in France as in other countries in the world [8–10].

Some studies have suggested an association between severe maternal morbidity in EP and substandard quality of care such as misdiagnosis, and diagnostic or therapeutic delays [6, 11]. Morbidity and complication events could be more sensitive criteria to evaluate quality of care than mortality [12]. We hypothesized that analysis of CEPSB might be a useful approach to audit medical practices and manage quality of care in gynecological units.

Women with EP represent an important part of the activity of gynecological emergency units and almost all women with CEPSB are hospitalized. In France, each hospital admission generates administrative and medical records in the French Medical Information System Program (MISP), or the “Programme de Médicalisation des Systèmes d’Information” (PMSI). This system was implemented in 1991 to measure medical activity and to finance health facilities in both private and public hospitals [13]. The hospital discharge database (HDD) is a standardized national database composed of discharge data from the MISP corresponding to hospital stays.

Routine data from HDD were utilized for multiple public health purposes. Various researchers have utilized HDD in epidemiology purposes, to measure disease incidence [14–16], rates of complications in healthcare settings [17] or to estimate severe maternal morbidity [18] of the second and third trimesters of pregnancy. However, few studies have measured morbidity of the first trimester of pregnancy from HDD [19]. Also, few studies highlight the role of medical routine data collection in the monitoring of quality of care [20].

The usefulness of routine data from HDD in different purposes such as epidemiological studies, quality of care, highlights of public health policies are based on diverse algorithms, constructed with the International Classification of Diseases and Health Related Problems (ICD) and/or the Common Classification of Medical Procedures (CCAM). Specific codes are available to describe the diagnosis and complications of CEPSB. The relevant question is whether the HDD could be used to detect CEPSB to manage clinical audits and quality of care.

The objective of this study was to propose a method to measure the validity of the hospital discharge database (HDD) to detect CEPSB among hospital stays in two gynecological units.

## Materials and methods

### Study population

The National Data Protection Authority (Commission Nationale de l’Informatique et des Libertés) approved the study on October 23, 2015 (n° 1859704). All data were fully anonymized before we accessed them.

The unit of analysis was the hospital stay, integrated an episode of hospital admission in gynecological unit. All hospital stays of women between 18 and 45 years old, admitted in two tertiary teaching gynecological units for acute pelvic pain or/and vaginal bleeding in 2012, were included. Hospital stays in a clinical context of post-partum, post-surgery or post-abortion, less than six weeks of the event, and of women with a history of chronic pelvic pain or whose pregnancy was beyond 15 weeks of amenorrhea, were excluded. During the study period, some women were hospitalized twice either for two different medical events (i.e., one CEPSB and one ovarian cyst rupture) or for the same pathology (i.e., one pelvic infection treated by antibiotics and a second hospitalization for laparoscopy). We counted each hospitalization as a hospital stays.

For our population, we recorded the specific clinical situation that justified each hospital stay. In case of several hospitalizations for the same patient, the administrative data (i.e., date of hospitalization or duration of hospitalization) helped us select the right medical record.

### Data sources

#### Hospital discharge database (HDD)

For each hospital stay, both administrative (age, sex, and type of admission) and medical (pathology and medical procedures) data are recorded in the HDD. The pathologies diagnosed during the hospital stay are coded with the 10^th^ edition of the ICD (ICD-10), according to the coding guidelines of medical activity, updated yearly by the Technical Agency for Information on Hospital Care [https://www.atih.sante.fr]. In the HDD, the main diagnosis retained is related to the health problem requiring the hospitalization and confirmed at the end of the hospital stay. Other diseases, complications or health risks that are diagnosed and treated during the hospital stay are recorded as associated diagnosis [21]. All procedures performed during the hospitalization are coded according to the French Common Classification of Medical Procedures (CCAM). The HDD data were coded at the end of the hospital stay by the senior gynecologist and controlled by a technician of the Medical Information Department.

#### Medical data

For each patient admitted or hospitalized in a health facility, a medical record is required. We collected “medical data” from the medical records of the women included in our study, and these data were considered as the gold standard. A CEPSB was defined as an EP (confirmed by histological analysis) and at least one of the following complications: (1) tubal rupture visualized by a tubal wall breach during the laparoscopy, (2) hemoperitoneum of 500cc or more measured by aspiration, (3) the presence of active bleeding. CEPSB cases were identified with clinical, biological and radiological criteria: a positive pregnancy test, uterine vacuity and/or an image of hematosalpinx, and/or pelvic effusion found by ultrasound. Laparoscopy was mandatory to confirm diagnosis.

All other medical situations (i.e., spontaneous abortion, ovarian cyst rupture, metrorrhagia of an intra-uterine pregnancy, uncomplicated EP) were considered as non-CEPSB cases.

The medical records were filed by the residents during the hospital stay. The medical data were collected by clinic research technicians in each hospital. The data management of the medical data was realized by one clinical researcher.

### Data processing and statistical analysis

#### Identification of CEPSB from HDD

Two algorithms were built to extract hospital stays related to CEPSB from the Medical Information Department.

The first algorithm (Table 1), called the “predefined algorithm” was constructed *a priori* from the coding guidelines of the MISP. This algorithm was composed of the following items: 1) administrative information related to inclusion criteria (sex: female; age: between 18 and 45 years; hospital stay during the year 2012 in gynecological unit); 2) diagnosed pathologies based on specific ICD-10 codes (EP for the main diagnosis and pregnancy-associated complications for the associated diagnosis); 3) specific procedures related to the EP treatment using the CCAM thesaurus (Table 1).

**Table 1:** Items integrated into the algorithms used to identify hospital stays for complicated ectopic pregnancy with severe bleeding (CEPSB) in the HDD.

	Algorithm operations
**Predefined algorithm**	Administrative data: Sexe (female), Age (between 18 and 45 years of age), period of analysis (2012), unit (gynecology unit). AND Diagnosis codes: **O00.1** (Tubal pregnancy) or **O00.0** (Abdominal pregnancy) or **O00.2** (Ovarian pregnancy) or **O00.8** (Other ectopic pregnancy) or **O00.9** (Ectopic pregnancy, unspecified). AND **O08.1** (Delayed or excessive haemorrhage following abortion and ectopic and molar pregnancy) or **K66.1** (Hemoperitoneum) or **Z51.3** (Blood transfusion) or **O08.3** (Shock following abortion and ectopic and molar pregnancy) or **O08.6** (Damage to pelvic organs and tissues following abortion and ectopic and molar pregnancy). AND Procedure codes: **JJFA001** (salpingectomy for ectopic pregnancy by laparotomy) or **JJFC001** (salpingectomy for ectopic pregnancy by laparoscopy) or **JQGA001** extraction of an abdominal ectopic pregnancy >13 week of amenorrhea by laparotomy) or **JJPC001** (salpingotomy + aspiration for ectopic pregnancy by laparoscopy) or **JJPA001** (salpingotomy + aspiration for ectopic pregnancy by laparotomy).
**Pragmatic algorithm**	Administrative data: Sexe (female), Age (between 18 ans 45 years of age), period of analysis (2012), unit (gynecology unit). AND Diagnosis codes: **O00.1** (Tubal pregnancy) or **O00.8** (Other ectopic pregnancy) or **O00.9** (Ectopic pregnancy, unspecified) or **O08.1** (Delayed or excessive haemorrhage following abortion and ectopic and molar pregnancy) or **O08.3** (Shock following abortion and ectopic and molar pregnancy). AND Procedure codes: **JJFA001** (salpingectomy for ectopic pregnancy by laparotomy) or **JJFC001** (salpingectomy for ectopic pregnancy by laparoscopy) or **JJPC001** (salpingotomy + aspiration for ectopic pregnancy by laparoscopy) or **JJJC002** (tubal expression for tubo-abdominal evacuation of ectopic pregnancy by laparoscopy) or **FELF001** (transfusion of packed red blood cells, of a volume superior of a half blood mass, under general or local-regional anaesthesia) or **FELF004** (transfusion of packed red blood cells, of a volume superior of a half blood mass, by adult).

EP, ectopic pregnancy; HDD, hospital discharge database

After this first data extraction, the association between the ICD-10 or CCAM codes and the data from hospital stays classified as CEPSB in the HDD was used to define the second algorithm. This “pragmatic algorithm” was constructed from codes with statistically significant associations and a positive likelihood-ratio > 4 (Table 1).

The pragmatic algorithm (Table 1) was composed of two items: 1) the diagnosed pathology, either the main or associated diagnosis with specific ICD-10 codes for EP; 2) EP-related procedures coded using the CCAM thesaurus.

#### Measure of the validity of the HDD to detect CEPSB

The validity of the HDD was measured by sensitivity, specificity and positive likelihood ratio values between the HDD and the medical data of each gynecological unit. Each hospital stay was considered as a specific clinical situation and was independently analyzed. The two algorithms were successively used to estimate the validity of the HDD.

True positives were defined as CEPSB cases that were accurately identified in the HDD with the algorithm. False positives were defined as non-CEPSB cases that were wrongly recorded as CEPSB in the HDD. False negatives were defined as CEPSB cases that were not recorded in the HDD as CEPSB. True negatives were all hospital stays for which CEPSB cases were listed neither in the patient’s record nor in the HDD.

The sensitivity was the probability of the HDD to correctly identify a CEPSB case. The specificity was the probability for the HDD to correctly identify a hospital stay for a non-CEPSB case. The positive likelihood-ratio was the odds ratio of the HDD to correctly identify a CEPSB case divided by (1- specificity).

We analyzed the false negative and the false positive cases according to the coding guidelines of medical activity to identify and classify coding errors.

The validity of each hospital’s HDD to identify CEPSB cases, measured by successively applying the predefined and pragmatic algorithms, were compared.

Confidence intervals were determined using an alpha risk of 5%. The statistical analysis was performed using SPSS version 22 (SPSS Inc., Chicago, IL) software.

## Results

A total of 385 hospital stays of patient, aged between 18 and 45 years old hospitalized for acute pelvic pain or/and metrorrhagia in 2012 were considered for inclusion. Fifteen of these (3.9%) had no medical records: nine from center A and six from center B and were excluded from analysis.

Of the remaining 370 hospital stays, 52 were defined by the medical data as CEPSB cases (28 in center A and 24 in center B) and 318 as non-CEPSB cases (Table 2).

**Table 2:** Characteristics of hospital stays of patient aged between 18 and 45 years old hospitalized for acute pelvic pain or/and vaginal bleeding in two gynecological units in 2012 (N = 370).

Characteristics of hospital stays	Center A	Center B	Total
**Total, n (%):**	230 (62.2)	140 (37.8)	370 (100.0)
For CEPSB cases, n (%)	28 (11.7)	24 (16.4)	52 (13.5)
For non-CEPSB cases, n (%)	202 (84.5)	116 (79.5)	318 (82.6)
Mean duration (days±1 SD)	2.11 (±1.7)	2.39 (±1.6)	2.21 (±1.7)
Mean age (years±1SD)	32.6 (±6.1)	31.0 (±7.3)	32.0 (±6.6)

The predefined algorithm applied to the HDD classified 12 stays as true positive cases of CEPSB, three as false positives, 40 as false negatives, and 315 as true negatives (Table 3).

**Table 3:** Validity of HDD to detect complicated ectopic pregnancies with severe bleeding (CEPSB) in two gynecological units in 2012 (N = 370).

	Positive tests, n	Sensitivity% [IC 95%]	Specificity% [IC 95%]	LR(+)[IC 95%]	False positives, n	False negatives, n
**Predefined algorithm**	15	23.1[11.6–34.5]	99.1[98.0–100.0]	24.5[7.2–83.8]	3	40
**Pragmatic algorithm**	50	63.5[50.4–76.5]	94.7[92.2–97.5]	11.9[7.2–19.7]	17	19

HDD, hospital discharge database; LR, likelihood ratio.

The pragmatic algorithm applied to the HDD classified 33 stays as true positive cases of CEPSB, 19 as false negatives, 17 as false positives, and 301 as true negatives (Table 3).

The hospital stays classified as false positives and false negatives were due to coding errors as shown in Table 4. Coding errors were related to the misuse of ICD-10 codes in 77.6% of cases, to the misuse of CCAM codes in 14.3% of cases and to administrative errors in 4.1% of cases. A misuse of ICD-10 could be for example: a complication such as "hemoperitoneum of more than 500cc" reported in the medical record that was not coded in the HDD with the specific ICD-10 code. The misuse of a CCAM code could be for example: the use of a nonspecific CCAM code “JJFC006” (total salpingectomy by laparoscopy) instead of the specific code for EP procedure “JJFC001” (total or partial salpingectomy for EP by laparoscopy). One hospital stay was not coded at all and one hospital stay for delivery was coded as a CEPSB.

**Table 4:** Description of coding errors in HDD for the 43 hospital stays classified as false positives (FP) and false negatives (FN).

Coding Errors		Total	Center A	Center B	P value*
Hospital stays classified FN (n)		46	21	25	
Hospital stays classified FP (n)		3	3	0	
1 coding error for the stay (n, %)		37	18 (48.6)	19 (51.4)	0.95
2 coding errors for the stay (n, %)		6	3 (50.0)	3 (50.0)	0.95
**Types of coding errors**					
False negatives	Incorrect code for principal diagnosis	3	2	1	0.61
	Absence of code for associated diagnosis	19	8	11	0.43
	Absence of code for complication	11	4	7	0.34
	Incorrect code for complication	2	1	1	1.00
	Absence of code for EP specific procedure	7	5	2	0.24
	Errors for administrative data	2	0	2	0.16
	Uncoded stays	1	1	0	1.00
	Use of ICD code for delivery	1	1	0	0.48
FP	Diagnosis codes used in excess	3	3	0	0.11

HDD, hospital discharge database; FN, false negative; FP, false positive

* Chi2 test or Fisher exact test.

The sensitivity of the HDD to detect hospital stays for CEPSB after applying the predefined algorithm was statistically different between the two hospitals (sensitivity in center A: 35.7%, in center B: 8.3%, p = 0.02) but not the specificity (specificity in center A: 98.5%, in center B: 100%, p = 0.56). The sensitivity and specificity of the HDD to detect CEPSB by applying the pragmatic algorithm were not statistically different between the two hospitals (sensitivity in center A: 71.4%, in center B: 54.2%, p = 0.20; specificity in center A: 95.0%, in center B: 94.0%, p = 0.68).

## Discussion

### Main findings

The principal finding of our study is that the HDD has a poor performance to detect cases of CEPSB. Our method, which involved the successive application of two algorithms, improved detection performance. Most of the errors in the two tertiary teaching gynecological units were due to incorrect coding practices, despite the presence of specific codes in ICD-10 and surgery procedures for CESPB.

### Strengths and limitations

We present a pragmatic method to improve detection of CEPSB with a view to evaluating quality of care. Our research took into account both coding guidelines and observed coding practices to build two algorithms applied successively as mentioned above. The first algorithm measured the gap between data from the HDD and the medical records, while the second improved the capability of the HDD to detect CEPSB. Our results justify the use of a rational approach, evaluating the inclusion or not of diagnosis and procedures according to coding guidelines on the one hand, and according to coding practices on the other.

Similarly to our study, other studies have found that the validity of an HDD varies according to the construction of the algorithm [22] and the pathology of interest [15]. For instance, a study on the detection of breast cancer found that the inclusion of procedure codes into the algorithm improved the specificity of HDD [23]. Other studies have used several algorithms to determine the reliability and the accuracy of the HDD, but without explaining how the algorithms were built [23, 24]. Our approach, which uses coding guidelines and miscoding based on the health providers’ coding practices, would appear to be relevant.

Our study optimized the validity of HDD in detecting CEPSB among our population by applying a pragmatic algorithm. The main difference between the predefined and pragmatic algorithm is the absence of a second mandatory ICD-10 code. This could increase false negatives and fail to identify complications of EP. Furthermore, the algorithms are likely to work accurately if the coding errors were eliminated. This is a critical situation since the performance of HDD depends on coding practices, medical quality data in hospitals [25, 26, 27], or recording data practices [13]. The improved validity of HDD using the pragmatic algorithm must be confirmed with another population at a regional and national level, taking into account different types of gynecological emergencies. Nevertheless, this is the first study to examine the performance of HDD to detect CEPSB with a view to evaluating quality of care [18, 22].

### Interpretation

The difficulty in detecting complicated medical situations with routinely collected HDD data throws into question its utilization as a database to identify near-miss cases to evaluate quality of care. Other studies have also assessed the use of complication codes in HDD. To evaluate the accuracy of diagnosis-type indicators for flagging complications, Quan *et al*. [17] attempted to select hospital stays based on complications to perform a clinical audit. They reported poor validity for some complications from HDDs for 1996 and 1997. In 1994, Hartz *et al*. [9] studied complications of coronary artery bypass surgery and data sources, and concluded that assessing quality of care using an HDD might be difficult. Our study comes to the same conclusion 20 years later: EP complications were under-coded and were unable to select hospital stays of interest. Although the healthcare providers’ understanding of HDD quality has improved over the last 10 years, coding practices of medical complications remains to be improved.

An analysis of coding errors may provide an understanding of miscoding practices and the reasons for misreporting medical information. As previously specified [18], data quality depends on medical information collected (diagnosis, procedures) and administrative data. In our study, coding errors were mainly due to the misuse of ICD-10 codes. Lombrail *et al* [28] discussed the lack of reliability of HDD in the use of uncertain diagnosis codes. Clinical difficulties in establishing an accurate diagnosis could explain the misuse of ICD-10 codes. Nevertheless, the diagnosis of CEPSB is confirmed by a surgical procedure that determines the final diagnosis during the hospital stay. Some coding errors could also be due to the use of two classifications, one for the diagnosis and one for the procedure. Other errors are due to the shortening or lengthing wording as previously described [29, 30].

In a study of the validity of severe maternal morbidity in HDD, Chantry *et al*. [18] reported that false positives are mainly due to the excessive use of diagnosis codes. They observed that some professionals used ICD-10 to describe a more severe medical situation than the real one. Furthermore, false negatives were mainly due to the use of non-specific procedure codes. Our study gave similar results though the proportion of coding errors was higher than in Chantry *et al*.’s study and the false negatives were due to inaccurate use of ICD-10 and procedure codes.

Chantry *et al*. [15] describe a link between the organization of medical information production and data quality in HDD. Computerization of medical data and information control in a simultaneous intermediary step were associated with an improvement in data quality. The authors show that various organizations of medical information production create differences in sensitivity and specificity of HDD in four hospitals. In our study, a significant difference was also observed between the performances of the two hospitals HDD, which could be explained by their organization.

This study is a first step for a possible program of clinical audit of pregnancy loss, such as EP, among gynecological units, since CEPSB is associated with substandard cares. Our study highlights that further improvements in coding practices need to be implemented. The implementation of a specific thesaurus with ICD-10 and procedure codes from the CCAM could help clinicians to standardize medical information collection, even if rare and severe medical situations could still be difficult to track [18, 26]. Improving the quality of HDD requires data quality assessment, including coding procedures for various clinical cases and staff training [18, 22, 29, 31, 32]. These different actions necessitate dedicated human resources [29] to ensure accurate routine care data.

These results reinforce the need for high standards in quality health data collection to be able to use HDDs for public health purposes [31].

## Conclusion

This validity study of HDDs to detect CEPSB highlights that HDD cannot currently be relied on to detect such cases. Nevertheless, the use of HDD could be a useful tool to identify near-miss cases and manage quality of care in gynecologic emergency units and services. The HDD approach to identify hospital stays for clinical audit should be adapted to take into account coding practices and data collection organization. Reliability of medical data must be improved with culture in medical information and data quality assessment among health care providers and health care systems.

## Supporting information

S1 Data set (XLSX)
